# Newly Diagnosed High-Risk Multiple Myeloma: Outcomes and Management

**DOI:** 10.1155/ah/6622365

**Published:** 2025-09-26

**Authors:** Fatma Zehra Yasar, Elan Gorshein

**Affiliations:** ^1^Department of Internal Medicine, Marmara University School of Medicine, Istanbul, Turkey; ^2^Section of Hematology, Department of Internal Medicine, Yale University School of Medicine, North Haven, Connecticut, USA

**Keywords:** bispecific antibodies, CAR-T therapy, cytogenetics, high-risk multiple myeloma

## Abstract

Multiple myeloma (MM) is a heterogeneous hematologic malignancy, with high-risk cytogenetic abnormalities (HRCAs) such as del(17p), t(4; 14), t(14; 16), and gain(1q) contributing to poor prognosis in approximately 20%–25% of newly diagnosed patients. These abnormalities are associated with aggressive disease, frequent relapses, and inferior progression-free and overall survival. This review explores the evolving therapeutic landscape for high-risk MM, focusing on induction strategies for both transplant-eligible and transplant-ineligible patients, the role of autologous stem cell transplantation (ASCT), and the use of consolidation and maintenance therapies. Emerging modalities such as bispecific antibodies and chimeric antigen receptor T-cell (CAR-T) therapies are examined, particularly in the context of their integration into earlier lines of treatment. Quadruplet induction regimens incorporating proteasome inhibitors, immunomodulatory drugs, and monoclonal antibodies have shown promise in improving outcomes and are becoming a cornerstone of frontline therapy. The review also emphasizes the potential of personalized, risk-adapted approaches based on cytogenetic profiling and minimal residual disease (MRD) monitoring. Ongoing clinical trials investigating the early use of CAR-T cells and bispecific antibodies may further transform the standard of care for patients with high-risk MM.

## 1. Introduction

Multiple myeloma (MM) is a blood cancer that originates from the malignant transformation of plasma cells derived from a single clone. It is the second most common hematological malignancy after non-Hodgkin lymphoma and is responsible for 20% of deaths from hematologic malignancies. The therapeutic landscape for MM has undergone impressive evolution in the past 2 decades, which has led to improvements in clinical outcomes and patient survival [1]. However, MM exhibits significant clonal heterogeneity, as the same treatment regimen may result in extended survival for some patients, whereas others may derive limited benefit. Fluorescence in situ hybridization (FISH) has been adopted as an important prognostic indicator in the era of more modern and effective treatments. High-risk disease is an evolving term in MM and may be characterized by the presence of del(17p), t(4; 14), t(14; 16), t(14; 20), chromosome 1 abnormalities, and/or extramedullary disease. These findings may be noted in approximately 20%–25% of patients at diagnosis and generally reflect an aggressive phenotype with more frequent relapses and lower rates of progression-free survival (PFS) as well as overall survival [2–4].

Minimal residual disease (MRD)-negative status has favorable prognostic value and is now incorporated into the International Myeloma Working Group (IMWG) response criteria [5, 6]. A randomized, phase II study that compared the addition of daratumumab to lenalidomide, bortezomib, and dexamethasone (D-RVd) to RVd in transplant-eligible, newly diagnosed multiple myeloma (NDMM) patients demonstrated that D-RVd significantly improved stringent complete response rates and MRD negativity for standard-risk cytogenetics but not for those with high risk [7]. Similar differences among standard-risk and high-risk patients were also noted in daratumumab-based regimens in the CASSIOPEIA and MAIA trials [8, 9]. On the other hand, carfilzomib-based regimens have been demonstrated to lead to durable complete remission (CR) and MRD-negativity, irrespective of the cytogenetic profile, which may lead to improved PFS and overall survival [10, 11]. The significance of MRD-negativity for disease prognosis is becoming increasingly clear, as it may abrogate high-risk cytogenetic features [12–14]. Indeed, MRD negativity is becoming a valid biomarker for PFS and overall survival [14].

In this review, we emphasize the evidence-based approaches currently recommended for the management of newly diagnosed high-risk MM patients.

## 2. High-Risk Definition and Prognostic Scores

The prognostic factors for MM patients can be categorized into three groups: disease biology and characteristics, host-related factors, and factors associated with tumor and host interactions. The factors related to disease characteristics are cytogenetic abnormalities and different staging systems. The Revised International Staging System (R-ISS) has recently been the preferred staging system for MM. The R-ISS supersedes earlier staging systems such as the Durie-Salmon Staging System, ISS, and IMWG-2014. The combination of beta-2 microglobulin, serum albumin, serum lactate dehydrogenase (LDH), and bone marrow FISH results incorporated into the R-ISS provides more comprehensive and robust insights than previous staging systems do. In contrast to the R-ISS, the R2-ISS incorporates the presence of 1q+ into the scoring system, with its calculation factoring in the prognostic relevance of concurrent chromosomal abnormalities. The R2-ISS represents a novel and straightforward prognostic algorithm. Compared with the R-ISS, the R2-ISS has demonstrated enhanced discriminatory power, particularly among the substantial cohort of patients with intermediate-risk NDMM [15]. The R2-ISS incorporates readily available and commonly used prognostic markers, and its additive calculation method readily accommodates the potential inclusion of new prognostic factors in the future [15] (Table 1: Staging Systems and High-Risk Cytogenetics in MM).

Cytogenetic abnormalities are categorized into primary translocations and secondary high-risk cytogenetic abnormalities (HRCAs), both of which shape clinical outcomes. Among primary translocations, t(11; 14) is generally associated with standard-risk disease, whereas t(4; 14), t(14; 16), and t(14; 20) are considered high-risk features, frequently cooccurring with secondary HRCAs such as del(17p), gain(1q), and TP53 mutations, which significantly worsen prognosis [16, 17]. Secondary abnormalities such as del(17p) (TP53 deletion), gain(1q), and del(1p32) are strong indicators of disease aggressiveness, with del(17p) frequently resulting in biallelic loss in high-risk MM [18]. Gain(1q) is highly associated with t(4; 14) (∼71%), and its presence further increases genomic instability and treatment resistance [17]. Additionally, MYC rearrangements, which often coexist with t(14; 16) or del(17p), are linked to advanced disease and plasma cell leukemia [16]. Patients with double-hit MM (≥ 2 HRCAs) and triple-hit MM (≥ 3 HRCAs) experience poor survival rates despite intensive treatment [19]. However, therapeutic advances, such as the use of BCL-2 inhibitors (venetoclax) for t(11; 14), proteasome inhibitors (bortezomib) for t(4; 14), and chimeric antigen receptor T-cell (CAR-T) or bispecific antibodies for ultrahigh-risk patients, are shaping personalized treatment strategies (Table 2: HRCAs and Prognostic Impact). Factors such as the atypical bone marrow plasma cell immunophenotype, increased plasma cell proliferative rate, plasmablastic morphology, increased number of circulating plasma cells, and extramedullary involvement can affect disease biology. Host-related factors such as age, performance status, and comorbidities are some of the elements linked to the prognosis of the disease. Extramedullary myeloma is linked to an unfavorable prognosis in both newly diagnosed and relapsing MM patients. Consequently, it holds clinical significance in enhancing the detection of extramedullary myeloma. Extramedullary myeloma is a diverse condition that affects approximately 15% of MM patients throughout their disease course and is related to poor prognosis [15].

## 3. Management of Newly Diagnosed High-Risk Patients

### 3.1. Induction

#### 3.1.1. Initial Treatment in Transplant Eligible Patients

##### 3.1.1.1. Bortezomib, Lenalidomide, and Dexamethasone (VRd)

The SWOG S0777 trial demonstrated the efficacy of VRd compared to lenalidomide plus dexamethasone (Rd), with approximately one-third of patients having high-risk disease (del(17p), t(14:16), and t(4; 14)). Importantly, in high-risk patients, treatment with VRd was associated with improved PFS (38 vs. 16 months), although this difference did not reach statistical significance, likely due to the limited number of high-risk patients in the study [20].

The IFM2008 trial further supported VRd efficacy, with high-risk cytogenetic features (del(17p) or t(4; 14)) observed in seven patients (27%). High-risk patients showed an estimated 3-year PFS of 86% (95% CI: 33%–98%), although this finding should be interpreted cautiously given the small sample size and limited statistical power to detect differences in this subgroup [21].

##### 3.1.1.2. Bortezomib, Cyclophosphamide, and Dexamethasone (VCd)

The EVOLUTION study compared VRd, VCd, and VDCR in newly diagnosed MM patients. VCd is considered a reasonable alternative to VRd, particularly for patients with a higher risk of renal toxicity. High-risk features, defined as del(13)/−13q14, t(4; 14), t(14; 16), −17p13, or hypodiploidy, were present in 17% (*n* = 24) of patients. Notably, high-risk patients achieved a 1-year PFS of 100%, suggesting that VCd may be particularly effective in this population despite the small sample size [22].

##### 3.1.1.3. Bortezomib, Thalidomide, Dexamethasone (VTd)

The IFM2013-04 trial demonstrated superior outcomes with VTd compared to VCd in transplant-eligible patients, with high-risk features defined as del(17p) and/or t(4; 14) [23]. The GIMEMA MM-BO2005 trial provided crucial insights into VTd's efficacy in high-risk disease. VTd consolidation was particularly beneficial for patients with t(4; 14), achieving superior 3-year PFS compared to thalidomide-dexamethasone (Td) consolidation (66% vs. 20%; *p*=0.001). Importantly, VTd consolidation appeared to overcome the adverse prognostic impact of t(4; 14), with nearly identical PFS curves regardless of t(4; 14) status (3-year estimates: 65% vs. 61%; *p*=0.936). In contrast, Td consolidation showed poor outcomes in t(4; 14)-positive patients (median PFS: 12 vs. 44 months in t(4; 14)-positive vs. negative patients; *p* < 0.0001), highlighting the specific benefit of proteasome inhibition in this high-risk subset [24].

##### 3.1.1.4. Daratumumab, Bortezomib, Lenalidomide, Dexamethasone (DVRd)

The GRIFFIN study compared DVRd with VRd in transplant-eligible patients. Among 30 patients (14%) with high-risk cytogenetics (del(17p), t(14:16), t(4; 14)), subgroup analysis revealed lower rates of stringent complete response (HR, 0.52; 95% CI, 0.09–2.90) and MRD-negativity (HR, 1.5; 95% CI, 0.32–6.99) compared to standard-risk patients. However, the limited number of high-risk patients precludes definitive conclusions, and larger studies are needed to confirm DVRd efficacy in this population [7].

The larger PERSEUS trial provided more robust evidence, with 21.7% of 709 patients having high-risk cytogenetics (del[17p], t[4; 14], t[14; 16]). Importantly, predetermined subgroup analyses demonstrated a consistent PFS advantage for DVRd over VRd across clinically relevant subgroups, including patients with high-risk cytogenetics, supporting the efficacy of this quadruplet regimen even in adverse-risk disease [25].

##### 3.1.1.5. Daratumumab, Bortezomib, Thalidomide, Dexamethasone (D-VTd)

The CASSIOPEIA trial randomized 1085 transplant-eligible patients to D-VTd versus VTd. High-risk cytogenetics (del[17p] or t[4; 14]) were present in 15% of the D-VTd group. Importantly, the benefit of D-VTd was attenuated in high-risk patients, with no significant advantage for achieving stringent CR (HR, 0.83; 95% CI, 0.42–1.66) or reducing risk of progression or death (HR, 0.67; 95% CI, 0.35–1.3). This suggests that high-risk patients may require alternative or more intensive treatment approaches beyond the addition of daratumumab to VTd [8].

##### 3.1.1.6. Daratumumab, Carfilzomib, Lenalidomide, Dexamethasone (Dara-KRd)

The MANHATTAN cohort included 41 patients, with 20 (49%) having high-risk features (1q+, t(4; 14), t(14; 16), t(14; 20), and/or 17p−). Notably, high-risk patients achieved MRD-negativity rates similar to standard-risk patients (odds ratio, 1.7; 95% CI, 0.36–8.6; *p*=0.50), suggesting that Dara-KRd may overcome adverse cytogenetic features [26].

The MASTER trial provided more robust evidence with 123 patients, where 37% had one and 20% had ≥ 2 HRCAs. MRD-negativity rates were maintained across risk groups: 78% (0 HRCAs), 82% (1 HRCA), and 79% (≥ 2 HRCAs). However, 3-year PFS demonstrated a clear risk stratification: 88% (no HRCAs), 79% (1 HRCA), and 50% (≥ 2 HRCAs), indicating that while deep responses are achievable, ultra-high-risk patients (≥ 2 HRCAs) still have inferior long-term outcomes despite intensive therapy [27].

##### 3.1.1.7. Daratumumab, Cyclophosphamide, Bortezomib, Lenalidomide, Dexamethasone (Dara-CVRd)

The OPTIMUM trial specifically targeted 107 transplant-eligible patients with ultra-high-risk disease, defined by 2 high-risk genetic markers (t(4; 14), t(14; 16), t(14; 20), gain(1q), del(1p), del(17p), and/or SKY92 gene expression profiles). This intensive pentuplet approach achieved MRD negativity in 41% postinduction and 64% postautologous stem cell transplantation (ASCT), demonstrating that even ultra-high-risk patients can achieve deep responses with sufficiently intensive therapy. The study provides proof-of-concept that treatment intensification through additional agents (cyclophosphamide addition) may be necessary for the most challenging patient population [28].

##### 3.1.1.8. Carfilzomib, Lenalidomide, and Dexamethasone (KRd)

The FORTE trial compared three treatment strategies in 474 transplant-eligible patients, with 34% having one and 26% having ≥ 2 HRCAs (t(4; 14), t(14; 16), del(17p), gain(1q), del(1p), or 1q amplification). High-risk outcomes demonstrated clear risk stratification: 4-year PFS was 60% (1 HRCA) versus 39% (≥ 2 HRCAs).

Importantly, KRd plus ASCT consistently outperformed other strategies in high-risk patients: 4-year PFS of 67% versus 57% (KRd alone) and 55% (KCd plus ASCT) for 1 HRCA patients, and 55% versus 31% and 33%, respectively, for ≥ 2 HRCA patients. KRd plus ASCT also achieved superior sustained MRD negativity irrespective of cytogenetic profile, establishing it as the preferred approach for high-risk disease [29].

##### 3.1.1.9. Isatuximab, Carfilzomib, Lenalidomide, and Dexamethasone (IsaKRd)

The IsKia trial randomized 302 transplant-eligible patients to IsaKRd versus KRd, with high-risk features (del(17p), t(4; 14), t(14; 16)) in 18%–19% and double-hit disease (≥ 2 HRCAs including 1q abnormalities) in 8%–9% of patients. IsaKRd demonstrated superior MRD negativity in high-risk patients: 76% versus 58% (1 HRCA) and 77% versus 53% (≥ 2 HRCAs). Notably, ultra-deep MRD negativity (10^−6^) was achieved in 72% and 77% of IsaKRd patients with 1 and ≥ 2 HRCAs, respectively, indicating profound disease control even in adverse-risk patients [30].

The GMMG-CONCEPT trial confirmed IsaKRd efficacy in an exclusively high-risk population (125 patients), achieving MRD negativity in 68% of transplant-eligible and 54% of transplant-ineligible patients, with sustained MRD negativity (≥ 1 year) in 63%. These consistent results across studies establish IsaKRd as highly effective for achieving deep responses in high-risk myeloma [31].

Several trials have assessed the efficacy of various induction regimens in transplant-eligible patients, with key studies summarized in Table 3 (Clinical Trials in Transplant-Eligible MM).

#### 3.1.2. Initial Treatment in Transplant Ineligible Patients

##### 3.1.2.1. Daratumumab, Lenalidomide, Dexamethasone (DRd)

The MAIA trial randomized 737 transplant-ineligible patients to DRd versus Rd, with high-risk cytogenetics (del(17p), t(14; 16), t(4; 14)) in ∼15% of patients. High-risk patients derived significant benefit from DRd, with median PFS of 45.3 versus 29.6 months (Rd) and median OS of 55.6 versus 42.5 months, respectively. These results establish DRd as an effective frontline option for transplant-ineligible high-risk patients, providing both progression-free and overall survival advantages [40].

##### 3.1.2.2. Bortezomib, Lenalidomide, and Dexamethasone (VRd-Lite)

The VRd-Lite study treated 53 transplant-ineligible patients with dose-reduced VRd. High-risk features were present in 6 patients (12%), though specific outcomes for this subgroup were not reported. This regimen represents a reasonable option for frail transplant-ineligible patients, particularly when considering renal function and tolerability [41].

##### 3.1.2.3. Daratumumab, Bortezomib, Melphalan, Prednisone (DVMP)

The ALCYONE trial randomized 706 transplant-ineligible patients to DVMP versus VMP, with high-risk cytogenetics (del(17p), t(4; 14), t(14; 16)) in 15%–17% of patients. Subgroup analysis showed a favorable trend for DVMP in high-risk patients (HR 0.78), though this did not reach statistical significance. The study suggests potential benefit for DVMP in high-risk transplant-ineligible patients, particularly those with R-ISS stage III disease (HR 0.75) [42].

##### 3.1.2.4. Isatuximab, Lenalidomide, Dexamethasone and Bortezomib (IsaVRd)

The BENEFIT trial compared Isa-VRd versus Isa-Rd in 270 transplant-ineligible patients with high-risk features (del(17p), t(4; 14), t(14; 16)) in 8%–10% of patients. The addition of bortezomib significantly improved MRD negativity (53% vs. 26%) and complete response rates (58% vs. 33%). Importantly, MRD benefits were consistent across subgroups, including high-risk patients, indicating that proteasome inhibitor addition is beneficial even in adverse-risk transplant-ineligible patients [43].

The IMROZ study randomized 446 transplant-ineligible patients to Isa-VRd versus VRd, with high-risk cytogenetics in 15%–19% of patients. While Isa-VRd improved MRD negativity and complete response rates in high-risk patients (55.5% vs. 40.9%; *p*=0.003), the PFS benefit was less pronounced in this subgroup (HR 0.97) and did not reach statistical significance. This suggests that high-risk transplant-ineligible patients may require alternative intensification strategies beyond anti-CD38 addition [44].

For transplant-ineligible patients, frontline therapy regimens have been evaluated in multiple studies, as outlined in Table 4 (Clinical Trials in Transplant-Ineligible MM).

### 3.2. Hematopoietic Stem Cell Transplantation (HCT)

#### 3.2.1. ASCT

ASCT remains a cornerstone of first-line therapy for transplant-eligible NDMM patients, demonstrating superior PFS compared to chemotherapy alone in randomized trials. For high-risk NDMM patients characterized by cytogenetic abnormalities such as del(17p), t(4; 14), and t(14; 16), ASCT provides significant benefits, though these patients typically have shorter PFS and OS than standard-risk patients and often require additional maintenance therapies and novel agents for optimal long-term disease control [50, 51]. Multiple landmark trials have established ASCT efficacy in high-risk disease. The IFM 2009 trial demonstrated that early ASCT was associated with superior PFS in high-risk patients, with a median PFS of 47.2 months in the ASCT group compared with 35 months in the VRd-alone group (HR 0.70, 95% CI 0.59–0.83) [45]. The DETERMINATION trial further confirmed these benefits, showing improved PFS in high-risk patients with ASCT versus VRd alone (median PFS 56 vs. 17 months; HR 1.99, 95% CI 1.21–3.26), though OS differences remain modest due to the effectiveness of salvage therapies, including second-line ASCT and novel agents [32]. Importantly, ASCT appears to enhance the depth of response in high-risk patients, with higher MRD negativity rates observed in the ASCT group (29.8% vs. 20% in the VRd-alone group; *p*=0.01), which serves as a strong predictor of long-term survival [45, 46]. This suggests that the intensive conditioning regimen may help overcome some of the resistance mechanisms associated with adverse cytogenetics.

The timing of ASCT is particularly crucial for high-risk patients. While the choice between early and delayed ASCT can depend on patient and physician preferences in standard-risk disease, the evidence favors upfront ASCT for high-risk patients. The IFM 2009 trial showed that high-risk patients experienced improved PFS when treated with early ASCT compared to delayed ASCT strategies, supporting the recommendation for upfront transplantation in this population [45, 46]. For high-risk patients, tandem ASCT represents a promising intensification strategy despite limited data support in the general population. Evidence supporting tandem ASCT in high-risk patients comes from the EMN02/HO95 study, which demonstrated that tandem ASCT significantly prolonged PFS compared to single ASCT in high-risk patients (HR 0.59, 95% CI 0.38–0.91; *p*=0.02) [52]. This study provides robust evidence that the graft-versus-myeloma effect and increased tumor cell kill achieved through tandem transplantation may be particularly beneficial for patients with adverse cytogenetic features. The improved outcomes observed in this high-risk subgroup support the consideration of tandem ASCT as a treatment intensification strategy, particularly when followed by appropriate maintenance therapies. Based on available data, proteasome inhibitor-based induction therapy should be administered to all high-risk patients, particularly those with the t(4; 14) translocation, as it may partially mitigate the adverse prognosis associated with this genetic abnormality. In transplant-eligible patients, early stem cell mobilization and collection should follow an initial therapy regimen of four cycles of VRd. For double- or triple-hit patients or those at ultra-high risk, incorporation of daratumumab into the core VRd regimen (D-VRd) should be considered, despite currently limited specific data in high-risk patients. This approach is founded on clinical experience with exceptionally poor prognosis cases among high-risk patients, where intensive treatment aimed at achieving the most profound possible response is crucial.

#### 3.2.2. Allogeneic Stem Cell Transplantation

Allogeneic HCT is a seldom-used approach beyond the confines of clinical trials. While it has the potential for a cure, it is burdened with significant treatment-related complications and a notable risk of mortality. Furthermore, no definitive evidence indicates improved overall survival compared with autologous HCT.

### 3.3. Consolidation

The EMN02/HO95 trial demonstrated improved PFS with VRd consolidation compared to no consolidation (58.9 vs. 45.5 months, HR 0.77, 95% CI 0.63–0.95), though OS benefits were not observed [33]. However, the BMT CTN 0702 trial found no significant benefit for consolidation therapy, showing similar PFS and OS rates between consolidation and maintenance-alone groups even at 6-year follow-up [53].

Current evidence suggests that additional consolidation therapy after ASCT is not routinely recommended for MM patients. However, for high-risk patients who may not achieve optimal depth of response with induction and ASCT alone, individualized consideration of consolidation therapy may be warranted, particularly in the context of clinical trials investigating novel agents or combinations.

### 3.4. Maintenance

#### 3.4.1. Immunomodulatory Agents

##### 3.4.1.1. Lenalidomide

Lenalidomide maintenance is the current standard of care for MM patients, providing significant PFS improvement, particularly in standard-risk patients. However, multiple studies demonstrate that high-risk patients derive less pronounced benefit and often require more aggressive maintenance strategies [34, 47, 48].

Several pivotal trials have evaluated lenalidomide maintenance outcomes by cytogenetic risk. In a phase III randomized trial by Palumbo et al., high-risk patients (del(17p), t(4; 14), del(13q), or t(14; 16)) showed nonsignificant trends toward improved PFS (HR 0.73, 95% CI 0.37–1.42) and OS (HR 0.79, 95% CI 0.31–2.01) with lenalidomide maintenance, contrasting with the significant benefit observed in standard-risk patients (HR 0.38, 95% CI 0.24–0.62) [49].

The Myeloma XI trial, the largest maintenance study to date with 1917 patients, confirmed this pattern. High-risk patients showed reduced benefit from lenalidomide maintenance, with particularly limited efficacy in ultra-high-risk patients (≥ 2 cytogenetic abnormalities). While lenalidomide maintenance improved outcomes across all subgroups, the magnitude of benefit was consistently smaller in high-risk disease, supporting the need for alternative or intensified maintenance approaches in this population [35].

##### 3.4.1.2. Bortezomib/Lenalidomide

A risk-adapted maintenance approach using bortezomib plus lenalidomide was evaluated in high-risk patients (25.1% of 1000 patients) with del(17p) and 1q amplification. This combination maintenance achieved a median PFS of 40.3 months and OS of 78.2 months in high-risk patients, demonstrating that dual-agent maintenance with a proteasome inhibitor and IMiD combination can improve outcomes in adverse-risk disease compared to lenalidomide alone [36].

#### 3.4.2. Proteasome Inhibitors

Bortezomib maintenance demonstrates particular efficacy in high-risk patients. The HOVON-65/GMMG-HD4 trial showed that bortezomib maintenance was superior to thalidomide maintenance specifically in patients with 17p deletion, achieving superior PFS (26 vs. 12 months, *p*=0.024) and 3-year OS (69% vs. 17%, *p*=0.028). This establishes proteasome inhibitor maintenance as especially important for high-risk cytogenetic subgroups [37].

Ixazomib shows particular benefit in high-risk patients. TOURMALINE-MM2 demonstrated improved PFS in high-risk patients (median 24 vs. 18 months, HR 0.69), while TOURMALINE-MM3 confirmed superior PFS rates (46% vs. 24%) in high-risk transplant-eligible patients. These findings support oral proteasome inhibitor maintenance specifically for high-risk disease [38, 54].

#### 3.4.3. Antibody Treatment

##### 3.4.3.1. Daratumumab

Daratumumab maintenance has emerged as a promising strategy for high-risk patients, with evidence from multiple landmark trials. The CASSIOPEIA trial demonstrated improved PFS with daratumumab maintenance across all subgroups, including high-risk patients (HR 0.39 for the high-risk subgroup), though the absolute benefit was less pronounced than in standard-risk disease [8].

The ALCYONE trial showed improved PFS and OS with daratumumab-based treatment in transplant-ineligible patients, but this improvement was not consistently observed in patients with high-risk cytogenetic features, highlighting the persistent challenge of treating adverse-risk disease even with novel antibody approaches [42].

More encouraging results came from the MAIA trial, which demonstrated PFS improvement with daratumumab plus lenalidomide-dexamethasone maintenance in both standard-risk (5-year PFS 53% vs. 29%) and high-risk patients (HR 0.57, 95% CI 0.32–1.03), though the benefit in high-risk patients did not reach statistical significance. The GRIFFIN and PERSEUS studies provided additional evidence for daratumumab maintenance benefit in high-risk transplant-eligible patients when combined with lenalidomide maintenance [40, 54, 55].

##### 3.4.3.2. Isatuximab

Isatuximab maintenance has shown promising results in high-risk patients across multiple studies. The GMMG-CONCEPT trial investigated isatuximab, carfilzomib, lenalidomide, and dexamethasone (Isa-KRd) followed by isatuximab maintenance in exclusively high-risk patients. MRD negativity rates were 67.7% and 54.2% in transplant-eligible and ineligible patients, respectively, with sustained MRD negativity maintained during isatuximab maintenance, indicating durable disease control [31].

The GMMG-HD7 trial randomized transplant-eligible patients to receive Isa-VRd versus VRd induction followed by isatuximab-lenalidomide versus lenalidomide maintenance. MRD negativity rates were significantly higher with isatuximab maintenance (50% vs. 36%, OR 1.82, 95% CI 1.33–2.48), suggesting that anti-CD38 maintenance may provide additional benefit beyond lenalidomide alone, and particularly in achieving deep responses [39].

The Phase 3 IMROZ study is evaluating isatuximab maintenance in transplant-ineligible patients, with results awaited. Current evidence suggests that isatuximab maintenance may be particularly valuable for maintaining deep responses in high-risk patients who achieve MRD negativity with intensive induction regimens [56].

##### 3.4.3.3. CAR-T and Bispecific Antibody Treatment (BsAbs)

CAR-T and bispecific antibodies represent promising emerging therapies for high-risk MM, with potential for deep, durable responses in patients with adverse cytogenetics. While established efficacy exists in relapsed/refractory disease, frontline applications are under active investigation [57, 58]. Early-phase studies demonstrate remarkable efficacy in high-risk newly diagnosed patients. Shi et al. evaluated dual anti-CD19 and anti-BCMA CAR-T cell therapy followed by lenalidomide maintenance after ASCT in 10 high-risk patients, achieving 100% ORR with 90% stringent complete response. Notably, 70% of patients maintained sustained MRD negativity for over 2 years at 42-month follow-up [58]. Du et al. confirmed these findings in 22 transplant-eligible patients with high-risk cytogenetics (del17p, t(4; 14), t(14; 16), or 1q21amp ≥ 4 copies), achieving 100% ORR and 95.5% stringent complete response with no PFS events reported [59]. These results suggest CAR-T therapy may overcome the poor prognosis associated with high-risk cytogenetic features.

Several pivotal trials are evaluating CAR-T integration into frontline therapy. CARTITUDE-5 compares VRd induction followed by cilta-cel versus VRd followed by maintenance in transplant-ineligible patients [60]. CARTITUDE-6 (EMagine) represents a paradigm-shifting trial comparing cilta-cel therapy directly with ASCT in transplant-eligible patients, potentially redefining the standard of care for newly diagnosed disease [61].

Bispecific antibodies offer off-the-shelf immunotherapy with promising early results. The MajesTEC-7 trial evaluates teclistamab plus daratumumab-lenalidomide versus DRd in transplant-ineligible patients [62]. The GEM-TECTAL trial specifically targets high-risk patients (del17p, t(4; 14), t(14; 16), and/or 1q amplification) with teclistamab-daratumumab and talquetamab-daratumumab combinations. For elranatamab, MagnetisMM-6 evaluates combination therapy versus DRd in transplant-ineligible patients [63], while MagnetisMM-7 compares elranatamab versus lenalidomide maintenance in MRD-positive post-ASCT patients [64]. The MonumenTAL-8 study investigates combination cilta-cel and talquetamab safety in high-risk patients.

These cellular and immunotherapies represent potentially transformative approaches for high-risk myeloma, offering the possibility of overcoming adverse cytogenetic features through enhanced immune recognition and elimination of malignant plasma cells. The integration of these modalities into frontline therapy may fundamentally change outcomes for patients with traditionally poor prognosis disease.

The role of CAR-T cell therapy and bispecific antibodies is rapidly evolving, with key clinical trials summarized in Table 5 (CAR-T and bispecific antibodies in MM).

## 4. Conclusion

MM is a heterogeneous hematological malignancy whose treatment paradigm continues to evolve rapidly. Patients with standard-risk disease may have sustained prolonged responses for years, with an overall survival that may exceed 10 years. Outcomes for higher-risk patients have advanced with the incorporation of immunomodulatory agents and monoclonal antibodies in the upfront setting; therefore, in addition to clinical trials, quadruplet-based therapy should be considered for newly diagnosed, high-risk disease. The introduction of bispecific antibodies and CAR-T cells for the treatment of naive patients is actively being evaluated, and future studies will hopefully translate to further improvements in this patient population.

## Figures and Tables

**Table 1:** Staging systems and high-risk cytogenetics in multiple myeloma.

Staging system		β2-microglobulin (mg/L)	Albumin (g/dL)	LDH level	High-risk cytogenetics	Other risk factors
R-ISS	Stage I	< 3.5	≥ 3.5	Normal	None	None
	Stage II	3.5–5.5	Any	Any	Any	Any
	Stage III	> 5.5	Any	Elevated	Any	Any

R2-ISS	Stage I	< 3.5	≥ 3.5	Normal	None	None
	Stage II	3.5–5.5	Any	Any	Any	Any
	Stage III	> 5.5	Any	Elevated	Plasma cell leukemia or extramedullary disease	Plasma cell leukemia or extramedullary disease

**Table 2:** High-risk cytogenetic abnormalities and prognostic impact.

Abnormality	Genes involved	Frequency (%)	Clinical features	Prognostic impact	Therapeutic considerations	Second HRCA
Primary abnormalities						
t(11; 14) (q13; q32)	CCND1	15–20	Lymphoplasmacytic morphology, affects apoptosis pathways	Standard risk	Responsive to BCL-2 inhibitors (venetoclax), proteasome inhibitors	None
t(4; 14) (p16; q32)	FGFR3, MMSET	10–15	Fewer lytic lesions, high extramedullary disease risk	High risk	Proteasome inhibitors (bortezomib) improve outcomes	Gain/Amp(1q) = 71%, Del(17p) = 11%
t(14; 16) (q32; q23)	MAF	3–5	Increased renal dysfunction risk, innate proteasome inhibitor resistance	High risk	Often combined with del(17p); poor response to standard therapy	Gain/Amp(1q) = 69%, Del(17p) = 22%, TP53mut = 14%, Del(1p) = 21%
t(14; 20) (q32; q12)	MAFB	1–3	Rare, aggressive phenotype	High risk	Similar to t(14; 16), poor outcomes with standard therapies	Any second HRCA = 60%, Del(17p) = 8%, t(4; 14) = 19%, t(14; 16) = 15%
Hyperdiploidy (HRD)	Multiple trisomies	45–50	Gains of odd-numbered chromosomes; slow disease progression	Standard risk	Better response to IMiD therapy (lenalidomide, pomalidomide)	None
Secondary abnormalities						
del(17p) (TP53 deletion/mutation)	TP53	10	Very poor survival, therapy resistance, high relapse rate	Ultra-high risk	Often requires aggressive therapy, CAR-T or bispecific antibodies may be promising	Biallelic loss = 4%
Gain(1q) or Amp(1q) (> 4 copies)	CKS1B, IL6R, ANP32E, BCL9	35–40	Increases disease instability, correlates with del(17p) and t(4; 14)	High risk	Associated with faster relapse and worse response to standard therapies	Del(1p) = 6%
del(1p32)	CDKN2C, FAM46C	10–15	High-risk subgroup, common with t(4; 14)	High risk	May benefit from novel immunotherapies	Biallelic loss = 3.4%
MYC rearrangement	MYC	15	Late-stage disease progression, found in plasma cell leukemia	High risk	No targeted therapy yet, linked to aggressive disease	Frequent co-occurrence with t(14; 16) and del(17p)
Double-hit MM	Any two high-risk abnormalities	14	Rapid progression, lower treatment response	Ultra-high risk	Combination therapy essential, including proteasome inhibitors, IMiDs, monoclonals	Various combinations of t(4; 14), t(14; 16), t(14; 20), del(17p), gain(1q), del(1p)
Triple-hit MM	Any three or more high-risk abnormalities	5–7	Extremely poor prognosis, fast relapse	Ultra-high risk	CAR-T, bispecifics, and early stem cell transplant recommended	Includes ≥ 3 of the high-risk lesions above

**Table 3:** Clinical trials in transplant-eligible multiple myeloma.

Reference	Study design	Study population	Regimen	High-risk definition	No. of high-risk patients	Primary end point	Overall outcome	Outcome of high-risk patients	≥ VGPR	MRD rates in high-risk patients
Voorhees, et al. [7]	Phase II randomized trial	207 transplant-eligible, newly diagnosed	D-RVd vs. RVd	del(17p), t(4; 14), t(14; 16), or amp(1q21)	32	sCR rate postconsolidation	sCR: 42.4% (D-RVd) vs. 32% (RVd)	sCR: 50% (D-RVd) vs. 11.1% (RVd) in high-risk.	90.9% (D-RVd) vs. 73.8% (RVd) achieved ≥ VGPR	62.5% (D-RVd) vs. 22.2% (RVd) achieved MRD negativity
Moreau, et al. [8]	Phase III randomized trial	1085 transplant-eligible, newly diagnosed	D-VTd vs. VTd	del(17p) or t(4; 14) cytogenetic abnormalities	168	sCR rate postconsolidation	D-VTd: Superior sCR (29% vs. 20%) and PFS (HR 0.47, *p*<0.0001)	Lower sCR rate in high-risk (D-VTd) vs. standard-risk.	83% (D-VTd) vs. 78% (VTd) achieved ≥ VGPR	64% (D-VTd) vs. 44% (VTd) achieved MRD negativity, lower in high-risk
Goicoechea, et al. [14]	Phase II trial	76 transplant-eligible newly diagnosed	KRdD	del(17p), t(4; 14), t(14; 16), or gain(1q21)	76	MRD negativity rate	71% MRD negativity; 100% ≥VGPR	71% MRD negativity; 100% ≥VGPR.	100% achieved ≥ VGPR	71% achieved MRD negativity
Durie, et al. [20]	Phase III randomized trial	525 newly diagnosed (no immediate transplant)	VRd vs. Rd	NA	NA	PFS, OS	PFS: VRd 43 months vs. Rd 30 months; OS: VRd 75 months vs. Rd 64 months	NA	70% (VRd) vs. 56% (Rd) achieved ≥ VGPR	NA
Roussel, et al. [21]	Phase II randomized trial	31 symptomatic	RVD induction followed by ASCT	del(17p), t(4; 14), or t(14; 16) cytogenetic abnormalities	7	PFS	PFS: 43 months; 3-year OS: 88%	NA	58% achieved ≥ VGPR	NA
Kumar, et al. [22]	Phase II randomized trial	150 newly diagnosed	VDC, VDR, VDCR	del(13)/13q14, t(4; 14), t(14; 16), del(17p13), Hypodiploidy	24	ORR	ORR: VDC 84%, VDR 85%, VDCR 74%; ≥VGPR: VDC 47%, VDR 42%, VDCR 35%	Adjusted 1-year PFS rates: VDCR: 83%, VDR: 68%,VDC: 97%,VDC-mod: 100%	47% (VDC), 42% (VDR), 35% (VDCR) achieved ≥ VGPR	NA
Moreau, et al. [23]	Phase III randomized trial	340 newly diagnosed	VTD vs. VCD	del(17p), t(4; 14), or t(14; 16) cytogenetic abnormalities	50	≥VGPR rate after induction therapy	≥VGPR: VTD 66% vs. VCD 56%, *p* = 0.04	NA	66% (VTD) vs. 56% (VCD) achieved ≥ VGPR	NA
Cavo, et al. [24]	Phase III randomized trial	390 newly diagnosed	VTD vs. TD as consolidation therapy post-ASCT	del(17p), t(4; 14), or t(14; 16) cytogenetic abnormalities	NA	PFS	Median PFS: VTD 60 months vs. TD 48 months, *p* = 0.01	NA	73% (VTD) vs. 61% (TD); *p* = 0.02 achieved ≥ VGPR	NA
Sonneveld, et al. [25]	Phase III randomized trial	1085 transplant-eligible	D-VRd vs. VRd	NA	NA	PFS	Median PFS: D-VRd (not reached) vs. VRd 61 months; HR for progression or death: 0.45 (*p*<0.001)	NA	84.1% (D-VRd) vs. 74.6% (VRd) achieved ≥ VGPR	64.2% (D-VRd) vs. 30.1% (VRd) achieved MRD negativity
Landgren, et al. [26]	Phase II nonrandomized trial	41 newly diagnosed	Dara-KRd	1q+, t(4; 14), t(14; 16), t(14; 20), and/or 17p−)	20	MRD negativity rate	71% MRD negativity; 100% ≥VGPR	NA	100% achieved ≥ VGPR	NA
Costa, et al. [27]	Phase II single-arm trial	123 newly diagnosed	Dara-KRd	t(4; 14), t(14; 16), del(17p), t(14; 20), or gain/amplification of 1q	48	MRD negativity rate	80% MRD negativity (<10^-5); 66% MRD <10^-6; 2-year PFS: 87%, OS: 94%	MRD negativity for 0, 1, 2+ HRCA: 78%, 82%, 79%; 2-year PFS for 2+ HRCA: 58%.	NA	79% MRD negativity in 2+ HRCA
Kaiser, et al. [28]	Phase II, multicenter	107 UHiR NDMM and PCL patients	Dara-CVRd induction, ASCT, extended Dara-VRd consolidation	≥2 genetic markers (t(4; 14), t(14; 16), t(14; 20), del(1p), del(17p), gain(1q)), SKY92 GEP, or PCL	107	PFS at 18 months	30-month PFS: 77%; OS: 83.5%; improvement vs. MyeXI comparator	UHiR patients: Sustained MRD negativity & high PFS with extended consolidation.	90% achieved VGPR or better	63.6% MRD negativity post-ASCT
Gay, et al. [29]	Phase II, randomized, open-label	474 newly diagnosed	KCyd, KRd with ASCT, or KRd without ASCT	NA	NA	PFS	KRd + ASCT improved PFS vs. KCyd + KRd without ASCT	NA	90% achieved VGPR or better	Higher MRD negativity in KRd arms
Leypoldt, et al. [31]	Phase II, multicenter	125 high-risk NDMM patients	Isa-KRd	HR defined as ISS II/III with del(17p), t(4; 14), t(14; 16), amp(1q21)	125	MRD negativity at consolidation	MRD negativity: TE 67.7%, TNE 54.2%; high sustainable deep responses	62.6% sustained MRD negativity for ≥1 year.	∼90% achieved VGPR	∼67% MRD negativity post-consolidation (TE)
Richardson, et al. [32]	Randomized phase 3	722 newly diagnosed, symptomatic, aged 18–65	RVD + ASCT vs RVD alone	High-risk cytogenetic profile: del(17p), t(4; 14) or t(14; 16) translocations	132	PFS	RVD + ASCT: 67.5 months, RVD alone: 46.2 months (HR 1.53, *p*<0.001)	High-risk PFS: 55.5 months (ASCT) vs 17.1 months (RVD).	NA	NA
Cavo, et al. [33]	Randomized, phase 3	1503 transplant-eligible, aged 18–65	Autologous HSCT vs. VMP	NA	1197	PFS	PFS: HSCT 56.7 months, VMP 41.9 months (HR 0.73, *p* = 0.0001)	PFS in high-risk not separately reported.	NA	NA
McCarthy, et al. [34]	Randomized, phase 3	460 transplant-eligible, aged 18–70	Lenalidomide maintenance post-ASCT vs. placebo	NA	NA	Time to disease progression, OS	Lenalidomide: Longer PFS vs. placebo (∼58% progression or death in 34-month follow-up)	HR for progression: 0.37 (*P* < 0.001).	Not explicitly stated but lenalidomide group showed improved survival	37% (lenalidomide) vs. 58% (placebo) had progression or death
Jackson, et al. [35]	Phase III Randomized, Open-Label, Multicenter	1917 patients (1137 lenalidomide, 834 observation)	Lenalidomide maintenance vs Observation	High-risk cytogenetic lesions: t(4; 14), del(17p), gain(1q), t(14; 16), t(14; 20)	219	PFS, OS	Lenalidomide: PFS improved, no OS improvement in overall population	PFS improved in high-risk, OS benefit seen only in transplant-eligible.	45% achieved ≥ VGPR	PFS improvement suggests possible MRD benefit
Joseph, et al. [36]	Prospective, single-Center, Observational	1000 patients	Lenalidomide, bortezomib, dexamethasone induction followed by risk-adapted maintenance therapy	High-risk cytogenetics: del(17p), t(4; 14), 1q amplification, t(14; 16)	220	PFS, OS	RVD induction + risk-adapted maintenance: Improved survival	Median PFS: 40.3 months, OS: 78.2 months in high-risk.	89.9% after induction; 33.3% achieved sCR after transplantation	PFS/OS improvements suggest MRD benefit
Sonneveld, et al. [37]	Randomized phase III Trial	827 patients	Induction with PAD followed by HDM and ASCT, maintenance with Bortezomib	High-risk defined by creatinine > 2 mg/dL, del(17p13), t(4; 14)	81	PFS, OS	Bortezomib: Improved PFS and OS vs. VAD	Bortezomib improved PFS (30 vs. 13 months) & OS (54 vs. 21 months) in high-risk patients with creatinine > 2 mg/dL.	76% (PAD arm) after induction	PFS/OS improvements suggest MRD benefit
Dimopoulos, et al. [38]	Phase III, double-blind, placebo-controlled	656 post-ASCT patients with response after high-dose melphalan	Ixazomib: Ixazomib 4 mg for 2 years or placebo	High-risk: Not specifically defined, but high-risk patients could be inferred based on response and pre-transplantation stage	NA	PFS	Ixazomib: PFS improved (26.5 vs. 21.3 months, HR 0.72, *p* = 0.0023)	Ixazomib: 27% serious adverse events vs 20% with placebo.	Ixazomib: Higher CR rate, improved deeper responses than placebo	MRD rates not specifically reported for high-risk patients
Goldshmidt, et al. [39]	Phase III, Open-Label, Multicenter, Randomized, Active-Controlled	NA	Isatuximab + VRd induction for 3 cycles, followed by maintenance	NA	NA	MRD negativity post-induction	Isa-VRd: Improved MRD negativity (50% vs. 36%) vs. VRd alone	Isatuximab group had significantly higher MRD negativity.	Not directly reported, but improved response in the isatuximab group	50% MRD negativity in isatuximab group vs 36% in control group

**Table 4:** Clinical trials in transplant-ineligible multiple myeloma.

Reference	Study design	Study population	Regimen	High-risk definition	No. of high-risk patients	Primary end point	Overall outcome	Outcome of high-risk patients	≥ VGPR	MRD rates in high-risk patients
Mina, et al. [3]	Pooled analysis of two phase II studies	72 high-risk, non-transplant eligible	KCd	del(17p), t(4; 14), t(14; 16), or high-risk gene expression profile	72	ORR and PFS	ORR: 88.9%; median PFS: 25 months	NA	66.7% achieved ≥ VGPR	NA
Facon, et al. [9]	Phase III randomized trial	737 non-transplant, newly diagnosed	D-Rd vs. Rd	del(17p), t(4; 14), or t(14; 16) cytogenetic abnormalities	63	PFS	Median PFS: D-Rd (not reached) vs. Rd (31.9 months); 30-month PFS: D-Rd 70.6%, Rd 55.6%	HR for progression/death in high-risk: 0.78 (95% CI, 0.38–1.61).	79.3% (D-Rd) vs. 53.1% (Rd) achieved ≥ VGPR	24.2% (D-Rd) vs. 7.3% (Rd) achieved MRD negativity
Facon, et al. [40]	Phase III, randomized	737 ineligible for ASCT, NDMM patients	D-Rd vs. Rd	NDMM, ineligible for ASCT due to age (≥65) or comorbidities	NA	PFS	Significant PFS and OS improvement in daratumumab group (HR 0.53 for PFS, HR 0.68 for OS)	Sustained OS and PFS improvement in daratumumab group.	∼90% (D-Rd) achieved VGPR	NA
O'Donnell, et al. [41]	Phase II single-Arm Study	50 transplant-ineligible, aged ≥65	RVD lite: Lenalidomide, bortezomib, dexamethasone	Deletion 17, t(4; 14), t(14; 16), t(14; 20)	6	ORR	ORR: 86%, VGPR+ 66%; Median PFS: 35.1 months; OS not reached	NA	66% achieved ≥ VGPR	NA
Mateos, et al. [42]	Randomized, phase 3	706 transplant-ineligible, aged ≥65	D-VMP with or without Dara	NA	NA	OS	HR for death: 0.60 (95% CI 0.46–0.80, *p*=0.0003); 36-month survival: D-VMP 78%, VMP 68%	NA	NA	NA
Leleu, et al. [43]	Randomized, phase 3	270 transplant-ineligible, aged 65–79	Isa-VRd with or without bortezomib	NA	NA	MRD negativity rate	Isa-VRd: 53%; IsaRd: 26% (OR 3.16, *p* < 0.0001)	Isa-VRd: 58%, IsaRd: 31% (*p* < 0.0001)	Isa-VRd: 53%, IsaRd: 26% (10−5) at 18 months achieved ≥ VGPR	58% at 18 months (Isa-VRd)
Facon, et al. [44]	Randomized, phase 3	446 transplant-ineligible, aged 18–80	Isa-VRd vs. VRd	NA	NA	PFS	Isa-VRd at 60 months: 63.2% (HR 0.60, *p* < 0.001) vs. VRd 45.2%	Isa-VRd: 74.7% vs VRd: 64.1% (*p*=0.01)	Isa-VRd: 55.5% vs. VRd: 40.9% (*p*=0.003) achieved ≥ VGPR	NA
Attal, et al. [45]	Randomized, phase 3	700 transplant-ineligible, aged ≤65	RVD followed by consolidation therapy. Transplant group received high-dose melphalan + ASCT	High-risk defined by t(4; 14), t(14; 16), or 17p deletion	90	PFS	Median PFS: RVD 36 months vs. Transplant 50 months (HR 0.65, *p* < 0.001)	PFS benefit in high-risk patients	48% (RVD) vs. 59% (Transplant) complete response rate	65% (RVD) vs. 79% (Transplant) MRD negativity
Perrot, et al. [46]	Randomized, phase 3	700 transplant-ineligible, aged ≥65	RVd: Lenalidomide, Bortezomib, dexamethasone	t(4; 14), t(14; 16), del(17p)	100	PFS	Median PFS: Transplant group 47.3 months, RVd alone 35.0 months (HR 0.70, *p* < 0.001)	Median PFS2: Similar in both groups; no OS difference	NA	MRD-negative status predicts longer PFS, PFS2, and OS
Palumbo, et al. [47]	Randomized, phase 3	459 transplant-ineligible, ≥ 65	MPR-R followed by lenalidomide maintenance	High-risk definition not specifically mentioned, but age >75 and Stage III considered high-risk	∼50% with stage III disease	PFS	MPR-R: PFS 31 months vs. 14 months (MPR), 13 months (MP)	Significant benefit in 65–75 years old (*p*=0.001 for age interaction)	77% response rate in MPR-R group	MPR-R showed significant PFS benefit
Attal, et al. [48]	Randomized, phase 3	614 transplant-ineligible, < 65	Lenalidomide maintenance after autologous stem-cell transplantation	High-risk not specifically defined, but cytogenetic abnormalities (t(4; 14), 13q deletion) considered high-risk	NA	PFS	Lenalidomide: PFS vs. placebo (41 vs. 23 months, HR = 0.50, *p* < 0.001)	Significant PFS improvement in all subgroups, including high-risk	69% achieved CR or ≥ VGPR post consolidation	MRD rates not explicitly stated but improved PFS noted
Palumbo, et al. [49]	Randomized phase III Trial	511 patients	Induction with VMPT followed by maintenance with VT for 2 years, compared to VMP	High-risk defined by age ≥75, ISS stage III, or specific cytogenetic abnormalities (e.g., t(4; 14), del(17p13))	138	PFS, OS, Time to next Therapy	VMPT-VT: Improved PFS and OS by ∼1 year vs. VMP	PFS improved in high-risk with VMPT-VT (HR = 0.58, *p* < 0.001)	38% (VMPT-VT) after induction, 56% at 3-year mark	Survival analysis suggests MRD benefit in high-risk patients with cytogenetic abnormalities

**Table 5:** CAR-T and bispecific antibodies in multiple myeloma.

Reference	Study design	Study population	CAR-T %/bispecific antibody	Regimen	High-risk definition	No. of high-risk patients	Primary end point	Overall outcome	Outcome of high-risk patients	≥ VGPR	MRD rates in high-risk patients
Garfall, et al. [57]	Pilot Study	1 patient with refractory MM, high-dose melphalan	CAR-T (CTL019)	High-dose melphalan + Auto HSCT + CTL019 infusion	CD19-positive minor population	NA	PFS	12-month CR with no progression	No measurable monoclonal protein, sCR achieved.	100% (no progression) achieved ≥ VGPR	99.95% no CD19 expression; MRD-negative at day 100
Shi, et al. [58]	Single-arm, exploratory study	10 high-risk, aged 18–65	CAR-T (BCMA + CD19)	PAD induction followed by ASCT, sequential anti-CD19 and anti-BCMA CAR T infusion, followed by lenalidomide maintenance	Extramedullary disease, chromosomal abnormalities (del(17p), t(4; 14), t(14; 16)), R-ISS stage III	10	PFS, OS, MRD negativity	Best response: 90% sCR, 10% CR; 70% sustained MRD negativity at 42-month follow-up	80% sCR, 20% VGPR at day 100 post-ASCT.	70% sustained MRD negativity for over 2 years, median PFS and OS not reached	NA
Du, et al. [59]	Single-arm, phase I	22 transplant-eligible, high-risk, aged 43–69	CAR-T (BCMA + CD19)	Induction with VRd followed by anti-BCMA and anti-CD19 CAR-T cells infusion	R-ISS II or III, del17p, t(4; 14), t(14; 16), 1q21 ≥ 4 copies, extramedullary disease, IgD type	22	ORR, sCR, MRD negativity, duration of Response (DOR), PFS	100% ORR, 95.5% sCR, 100% MRD negativity at Months 1, 6, 12	100% MRD negativity across all dose levels.	100% ORR, 95.5% sCR (Cilta-cel arm)	100% MRD negativity at Month 1, 6, and 12
Dytfeld, et al. [60]	Randomized, phase 3	650 transplant-ineligible, high-risk	CAR-T (BCMA + CD19)	VRd induction followed by cilta-cel (CAR-T) or Rd maintenance	R-ISS II or III, del(17p), t(4; 14), t(14; 16), 1q21 ≥ 4 copies, extramedullary disease, IgD type	650	PFS, MRD-negative CR, OS	100% ORR, 95.5% sCR, sustained MRD negativity at Months 1, 6, 12	100% MRD negativity.	NA	100% MRD negativity at Month 1, sustained at 6 and 12 months
Broijl, et al. [61]	Randomized, phase 3	750 transplant-eligible, newly diagnosed	CAR-T (BCMA + CD19)	DVRd induction followed by CAR-T or ASCT and consolidation therapy	High-risk includes R-ISS II or III, del(17p), t(4; 14), t(14; 16), 1q21 ≥ 4 copies, extramedullary disease, IgD type, LDH > upper limit of normal, mSMART3.0 high-risk features	750	PFS, MRD-negative CR sustained for ≥ 12 months	Cilta-cel: 100% ORR, 95.5% sCR; ASCT PFS ongoing, initial favorable response	Cilta-cel: 100% MRD negativity, 80% sCR.	100% ORR, 95.5% sCR (Cilta-cel arm)	MRD-negative CR sustained ≥ 12 months, assessed by next-gen sequencing at 10−5
Grosicki, et al. [63]	Randomized, phase 3	∼646 transplant-ineligible, aged ≥65	Bispecific Antibody (elranatamab + daratumumab)	EDR vs. DRd	High-risk includes age ≥ 65 or < 65 with comorbidities impacting transplant eligibility	∼646	PFS, MRD at 12 months	EDR: 100% ORR, improved response duration, favorable safety	EDR arm: 90% MRD negativity, sustained > 1 year.	100% ORR (EDR)	90% MRD-negativity (EDR) at 12 months
Manteca, et al. [64]	Randomized, phase 3	∼700 post-transplant NDMM patients	Bispecific Antibody (elranatamab)	Elranatamab vs. Lenalidomide	High-risk defined by response post-ASCT, with or without consolidation	∼700	PFS, MRD at 12 months	Pending results	Pending results.	Pending results	Pending results

## Nomenclature

HR: High-risk
MM: Multiple myeloma
NDMM: Newly diagnosed multiple myeloma
PFS: Progression-free survival
OS: Overall survival
ASCT: Autologous Stem Cell Transplantation
MRD: Minimal Residual Disease
IMWG: International Myeloma Working Group
R-ISS: Revised International Staging System
R2-ISS: Second Revision of the International Staging System
HRCAs: High-risk cytogenetic abnormalities
PI: Proteasome Inhibitor
IMiD: Immunomodulatory Drug
CAR-T: Chimeric antigen receptor T-cell therapy
BsAb: Bispecific antibody
CR: Complete Response
VGPR: Very Good Partial Response
ORR: Overall Response Rate
BCMA: B-Cell Maturation Antigen
LDH: Lactate Dehydrogenase
CASSIOPEIA: Phase 3 Trial for Daratumumab-VTd vs VTd
GRIFFIN: Phase 2 Trial for Daratumumab-VRd vs VRd
PERSEUS: Phase 3 Trial for Daratumumab-VRd vs VRd
MAIA: Phase 3 Trial for Daratumumab-Rd vs Rd
CARTITUDE: CAR-T Therapy Trial for Myeloma
ALCYONE: Phase 3 Trial for Daratumumab-VMP vs VMP
FORTE: Phase 2 Trial for KRd vs ASCT-Based Treatment
HOVON: Dutch-Belgian Hemato-Oncology Group Study
GMMG: German-Speaking Myeloma Multicenter Group
IFM: Intergroupe Francophone du Myélome
EMN: European Myeloma Network
DVRd: Daratumumab + Bortezomib + Lenalidomide + Dexamethasone
D-VTd: Daratumumab + Bortezomib + Thalidomide + Dexamethasone
D-KRd: Daratumumab + Carfilzomib + Lenalidomide + Dexamethasone
VTD: Bortezomib + Thalidomide + Dexamethasone
VRd: Bortezomib + Lenalidomide + Dexamethasone
KRd: Carfilzomib + Lenalidomide + Dexamethasone
VMP: Bortezomib + Melphalan + Prednisone
Rd: Lenalidomide + Dexamethasone
DVMP: Daratumumab + Bortezomib + Melphalan + Prednisone
Isa-KRd: Isatuximab + Carfilzomib + Lenalidomide + Dexamethasone
CI: Confidence interval
FISH: Fluorescence in situ hybridization
GIMEMA: Gruppo Italiano Malattie Ematologiche dell'Adulto
HCT: Hematopoietic cell transplantation
IMROZ: Phase 3 Trial Evaluating Isatuximab-KRd in Newly Diagnosed Myeloma
MAGNETISMM: Clinical Trial Investigating Elranatamab in Multiple Myeloma
MANHATTAN: Study of KRd with Daratumumab in Myeloma
MASTER: Clinical Trial Investigating MRD-Adapted Therapy in Myeloma
MYC: Myelocytomatosis Oncogene
NEWLY: Newly Diagnosed Myeloma
OPTIMUM: Trial Investigating Quadruplet Therapy for High-Risk Myeloma
ORCID: Open Researcher and Contributor ID
SWOG: Southwest Oncology Group
TOURMALINE: Study Evaluating Ixazomib in Myeloma
VAMP: Vincristine, Adriamycin, and Methylprednisolone
VCD: Bortezomib, Cyclophosphamide, and Dexamethasone
VDCR: Bortezomib, Dexamethasone, Cyclophosphamide, and Lenalidomide
VMCP: Bortezomib, Melphalan, Cyclophosphamide, and Prednisone
VMPT: Bortezomib, Melphalan, Prednisone, and Thalidomide
